# Time Courses of Inflammatory Markers after Aneurysmal Subarachnoid Hemorrhage and Their Possible Relevance for Future Studies

**DOI:** 10.3389/fneur.2017.00694

**Published:** 2017-12-22

**Authors:** Anke Höllig, Birgit Stoffel-Wagner, Hans Clusmann, Michael Veldeman, Gerrit A. Schubert, Mark Coburn

**Affiliations:** ^1^Department of Neurosurgery, University Hospital RWTH Aachen, Aachen, Germany; ^2^Department of Clinical Chemistry and Clinical Pharmacology, University Hospital Bonn, Bonn, Germany; ^3^Department of Anesthesiology, University Hospital RWTH Aachen, Aachen, Germany

**Keywords:** inflammatory response, subarachnoid hemorrhage, early brain injury, delayed cerebral ischemia, observational research

## Abstract

**Object:**

Aneurysmal subarachnoid hemorrhage triggers an intense inflammatory response, which is suspected to increase the risk for secondary complications such as delayed cerebral ischemia (DCI). However, to date, the monitoring of the inflammatory response to detect secondary complications such as DCI has not become part of the clinical routine diagnostic. Here, we aim to illustrate the time courses of inflammatory parameters after aneurysmal subarachnoid hemorrhage (aSAH) and discuss the problems of inflammatory parameters as biomarkers but also their possible relevance for deeper understanding of the pathophysiology after aSAH and sophisticated planning of future studies.

**Materials and methods:**

In this prospective cohort study, 109 patients with aSAH were initially included, *n* = 28 patients had to be excluded. Serum and—if possible—cerebral spinal fluid samples (*n* = 48) were retrieved at days 1, 4, 7, 10, and 14 after aSAH. Samples were analyzed for leukocyte count and C-reactive protein (CRP) (serum samples only) as well as matrix metallopeptidase 9 (MMP9), intercellular adhesion molecule 1 (ICAM1), and leukemia inhibitory factor (LIF) [both serum and cerebrospinal fluid (CSF) samples]. Time courses of the inflammatory parameters were displayed and related to the occurrence of DCI.

**Results:**

We illustrate the time courses of leukocyte count, CRP, MMP9, ICAM1, and LIF in patients’ serum samples from the first until the 14th day after aSAH. Time courses of MMP9, ICAM1, and LIF in CSF samples are demonstrated. Furthermore, no significant difference was shown relating the time courses to the occurrence of DCI.

**Conclusion:**

We estimate that the wide range of the measured values hampers their interpretation and usage as a biomarker. However, understanding the inflammatory response after aSAH and generating a multicenter database may facilitate further studies: realistic sample size calculations on the basis of a multicenter database will increase the quality and clinical relevance of the acquired results.

## Introduction

Early brain injury (EBI) after aneurysmal subarachnoid hemorrhage (aSAH) repeatedly has been associated with a rise of inflammatory parameters measured in serum and cerebrospinal fluid (CSF). However, results are ambiguous concerning the prognostic value of inflammatory serum or CSF parameters. Several studies have proposed single inflammatory markers to be predictive for delayed cerebral ischemia (DCI) or outcome (1–8). Thus far, due to the wide individual and interindividual range, inflammatory parameters are limited serving as biomarkers to detect complications as DCI or to predict outcome on an individual basis. However, current data are heterogeneous and partly conflicting as there is no consensus, e.g., on the compartment for sample acquisition (serum or CSF or extracellular fluid—ECF—acquired *via* microdialysis) or the time point for sampling. Neuroinflammation is a major aspect of EBI. However, it remains a challenge to differentiate beneficial from devastating inflammatory components. Furthermore, the inflammatory response to the initial injury consists of various components such as cellular reactions as in terms of microglia activation (9), induction of cytokines and chemokines (1, 8, 10), leukocyte–endothelial cell interactions (11), and modulation of receptor expression (12). A complex pattern of immunoreaction is initiated after aSAH. Besides the inflammatory activation, there are various other pathophysiological reactions, which are initiated after SAH; such as microthrombosis, cortical spreading depolarization, microvasospasm, and blood–brain barrier breakdown, altogether resulting in dysfunction of the cerebral microcirculation (13). In turn, microcirculatory dysfunction may aggravate the inflammatory activation. Thus, the interactions of the initiated reactions hamper the interpretation of a single parameter’s dynamics. There is a vast amount of data on inflammatory parameters after aSAH usually in search for biomarkers predicting DCI. However, none of the examined parameters has found its way into clinical routine. This is most likely due to the abovementioned enormous individual and interindividual range and the low specificity of inflammatory parameters in general. Here, we present our data from a prospective observational study to illustrate the courses of various inflammatory parameters after aSAH. We hypothesize that monitoring of inflammatory parameters during the acute phase after aSAH does not allow an individual risk estimation (e.g., regarding the occurrence of DCI). However, detailed documentation of a vast amount of parameters may increase the understanding of the pathophysiological reaction after aSAH. Sophisticated knowledge of the pro-inflammatory reaction after aSAH and its interactions may possibly allow identification of specific anti-inflammatory therapeutic approaches.

Taken together, we aim to demonstrate that individual risk estimation for secondary complications after aSAH using inflammatory biomarkers is not promising. Instead, we vote for a multicenter database clarifying the courses and interactions of inflammatory parameters after aSAH to provide deeper insight of the pathophysiology of EBI.

## Materials and Methods

The prospective cohort study was approved by the local ethics committee (Ethikkommission an der Medizinischen Fakultät der Rheinischen Friedrich-Wilhelms-Universität Bonn, Germany; EK 199/08), written informed consent according to the Declaration of Helsinki was obtained from patients or legal representatives. Data from this study have already been published elsewhere (8, 14, 15). A total of 109 consecutive patients with aSAH were screened for eligibility within a 21-month period. Patients were not eligible if they were younger than 18 years, enrolled in other clinical trials, admitted more than 12 h after onset or if informed consent could not be obtained. Due to these criteria, 28 patients were excluded (*n* = 15 declined participation or data were lost, *n* = 9 were delayed admitted, *n* = 4 participated in another trial). We documented demographic, clinical, laboratory, and radiological data within an anonymized file history. Inflammatory parameters in serum and CSF were assessed at days 1, 4, 7, 10, and 14 after aSAH. CSF was tested in patients with necessity for CSF drainage mostly due to hydrocephalus (*n* = 48). Sample acquisition regularly took place in line with routine diagnostic (between 5:00 and 6:00 a.m.).

### Sample Processing

Blood and CSF samples were centrifuged for 10 min at 2,000 × *g* before processing. Samples were analyzed at the Department of Clinical Chemistry and Clinical Pharmacology, University of Bonn, either as part of routine diagnostics [leukocyte count (G/L), determination of C-reactive protein (CRP, mg/L), and interleukin 6 (IL6, pg/mL)] or for study purposes, by means of enzyme-linked immunofluorescence assays for determination matrix metallopeptidase 9 (MMP9, ng/mL), intercellular adhesion molecule 1 (ICAM1, ng/mL), and leukemia inhibitory factor (LIF, pg/mL) in serum samples (all assays purchased by IBL International GmbH, Hamburg, Germany).

### Analysis

Courses of serum parameters (all patients included), additionally CSF parameters and the corresponding serum parameters of the patients with CSF drainage were plotted dependent on time of sample acquisition. Graphs were acquired using GraphPad Prism^®^ 6. Furthermore, courses were analyzed using the Mann–Whitney *U* test. Occurrence of DCI was used as grouping variable to illustrate the usual approach in search of a biomarker indicating the occurrence of DCI. Therefore, DCI was defined as secondary neurologic worsening (increase in modified National Institutes of Health Stroke Scale >2 points). Furthermore, clinical improvement after induced hypertension and new cerebral ischemia or perfusion deficit confirmed by cranial computed tomography or magnetic resonance imaging (in case of consciousness or sedation or persistent neurologic deficit) were defined as indicators of DCI (if not explained by other causes such as embolism during angiography). Other relevant known causes such as infection, seizure, and metabolic, or electrolyte disturbances were excluded before determination of DCI. Tests were carried out using SPSS^®^ 21.

## Results

Baseline characteristics of the patients included is presented as supplemental data table (see Table S1 in Supplementary Material). Infectious complications occurred regularly with *n* = 8 (9.9%) at day 1, *n* = 27 (33.3%) at day 4, *n* = 38 (46.9%) at day 7, *n* = 36 (44.4%) at day 10, and *n* = 26 (32.1%) at day 14.

### Interleukin 6

**Figure 1:**
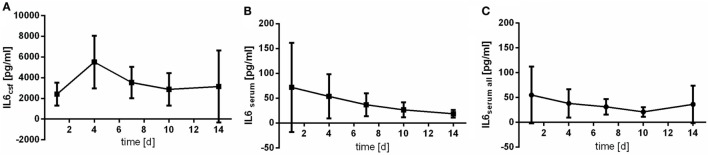
Time courses of interleukin 6 (IL6) in cerebrospinal fluid (CSF) samples [**(A)**; IL6csf], serum samples of the patients with CSF samples [**(B)**; IL6serum], and all serum samples available [**(C)**; IL6serum all] and their 95% confidence interval are displayed. Differences between nadir and peak values assessed *via* Wilcoxon Rank Test: IL6serum: day 14 vs. day 1: *p* = 0.029; IL6csf: day 1 vs. day 4: *p* = 0.184.

Serum course of IL6 differs from the pattern observed in CSF samples (Figures 1B,C). The peak of the measured serum level represents values obtained at day 1 after aSAH, whereas course of IL6 in CSF peaks somewhat later (at day 4) (Figure 1A). CSF values are far higher than serum levels (up to 10-fold). Of note, confidence intervals especially for serum values are huge due to the enormous variability of individual values.

### Matrix Metallopeptidase 9

**Figure 2:**
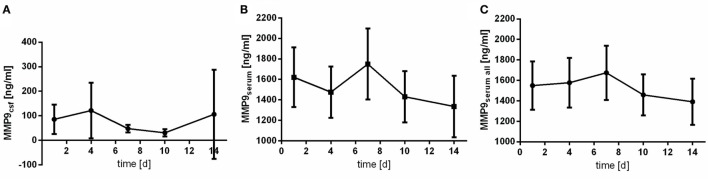
Time courses of matrix metallopeptidase 9 (MMP9) in cerebrospinal fluid (CSF) samples [**(A)**; MMP9csf], serum samples of the patients with CSF samples [**(B)**; MMP9serum], and all serum samples available [**(C)**; MMP9serum all] and their 95% confidence interval are displayed. Differences between nadir and peak values assessed *via* Wilcoxon Rank Test: MMP9serum: day 14 vs. day 7: *p* = 0.103; MMP9csf: day 10 vs. day 4: *p* = 0.001.

Matrix metallopeptidase 9 peaks earlier in CSF samples (at day 4) than in serum samples (at day 7) (Figure 2). Contrary to IL6, MMP9 is found in patients’ serum in far higher concentrations then in CSF.

### Intercellular Adhesion Molecule 1

**Figure 3:**
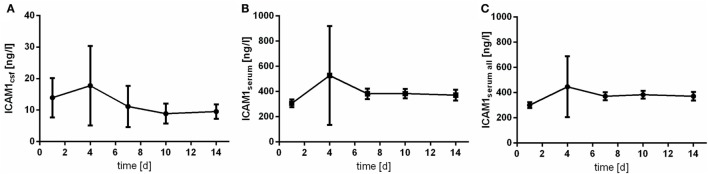
Time courses of intercellular adhesion molecule 1 (ICAM1) in cerebrospinal fluid (CSF) samples [**(A)**; ICAM1csf], serum samples of the patients with CSF samples [**(B)**; ICAM1serum], and all serum samples available [**(C)**; ICAM1serum all] and their 95% confidence interval are displayed. Differences between nadir and peak values assessed *via* Wilcoxon Rank Test: ICAMserum: day 1 vs. day 4: *p* = 0.001; IL6csf: day 10 vs. day 4: *p* = 0.906.

Intercellular adhesion molecule 1 is detectable in all of the acquired serum samples. In CSF samples, lower limit of detection (6.3 ng/mL) was not reached in 45.8% (*n* = 22) of the samples. Highest value of CSF ICAM1 expression was 56.8 ng/mL. Of note, the peak of the course is observed at day 4 after aSAH (Figure 3). However, variability especially at day 4 is extraordinary high.

### Leukemia Inhibitory Factor

**Figure 4:**
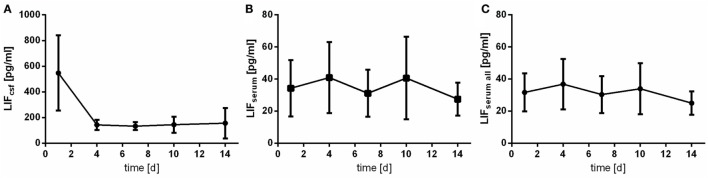
Time courses of leukemia inhibitory factor (LIF) in cerebrospinal fluid (CSF) samples [**(A)**; LIFcsf], serum samples of the patients with CSF samples [**(B)**; LIFserum], and all serum samples available [**(C)**; LIFserum all] and their 95% confidence interval are displayed. Differences between nadir and peak values assessed *via* Wilcoxon Rank Test: LIFserum: day 14 vs. day 4: *p* = 0.081; IL6csf: day 4 vs. day 1: *p* < 0.0001.

Leukemia inhibitory factor was detected reliably in patients’ CSF samples (LOD: 3.13 pg/mL). The course of LIF measured in CSF differs a lot from the one measured in serum samples (Figure 4): in CSF, a very early peak is observed followed by a rapid drop (Figure 4A). The assessed CSF levels are quite homogeneous demonstrated by low SDs. Serum levels of LIF are far lower (up to 10-fold) (Figures 4B,C).

### CRP and Leukocyte Count

**Figure 5:**
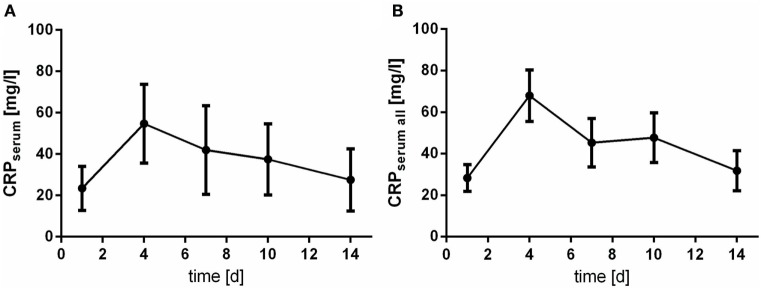
Time courses of C-reactive protein (CRP) in serum samples of the patients with cerebrospinal fluid samples [**(A)**; CRPserum] and all serum samples available [**(B)**; CRPserum all] and their 95% confidence interval are displayed. Differences between nadir and peak values assessed *via* Wilcoxon Rank Test: CRP: day 1 vs. day 4: *p* < 0.0001.

**Figure 6:**
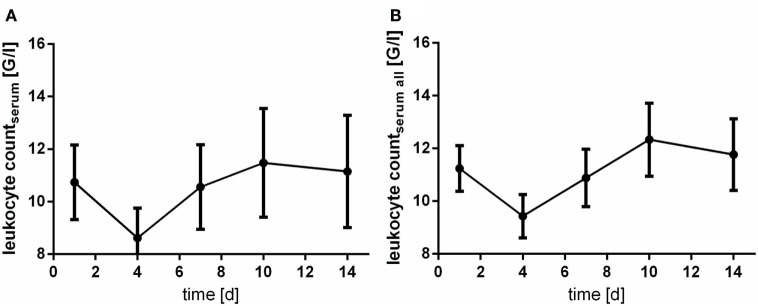
Time courses of leukocyte count in serum samples of the patients with cerebrospinal fluid samples [**(A)**; leukocyte countserum] and all serum samples available [**(B)**; leukocyte countserum all] and their 95% confidence interval are displayed. Differences between nadir and peak values assessed *via* Wilcoxon Rank Test: leukocyte count: day 4 vs. day 10: *p* < 0.0001.

The levels of CRP and leukocyte count were only assessed in serum samples. Of note, courses of CRP and leukocyte count act almost inversely. At day 4 after aSAH CRP levels peaks (Figure 5), whereas leukocyte count shows its nadir (Figure 6).

### Courses of Inflammatory Parameters Related to DCI

**Figure 7:**
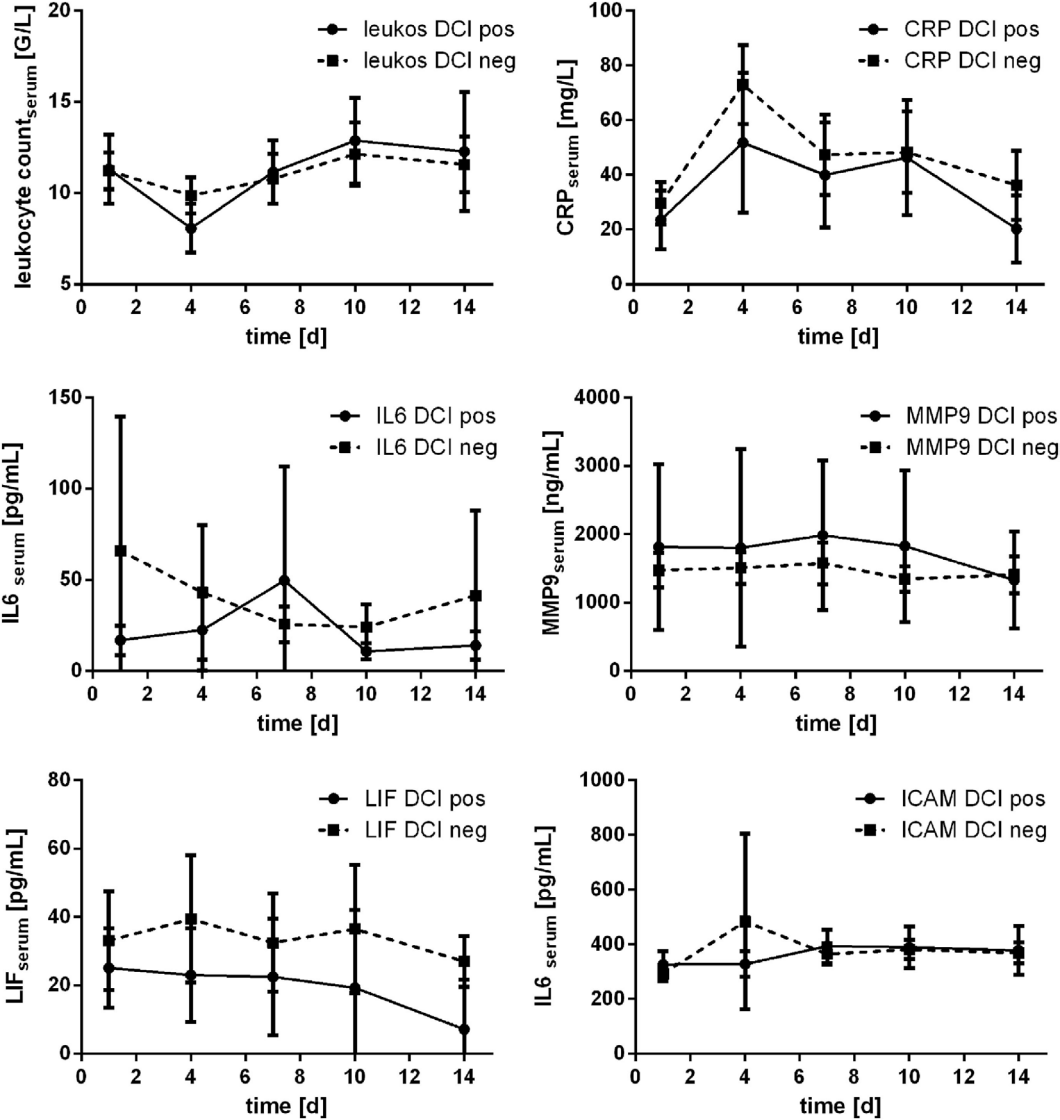
Courses of serum inflammatory parameters related to occurrence of delayed cerebral ischemia (DCI) (±95% confidence interval).

In 18 patients (22.2%), occurrence of DCI was detected (day 3: *n* = 1; day 4: *n* = 5; day 6: *n* = 2; day 7: *n* = 8; day 9: *n* = 1; day 10: *n* = 1). Comparing the courses of the assessed parameters related to occurrence of DCI, no significant differences were detected (Mann–Whitney *U* test). Marginal significance was only seen for leukocyte count at day 4 (*p* = 0.054). Courses are provided in Figure 7.

### Courses of Inflammatory Parameters Related to Fisher Scale Score

**Figure 8:**
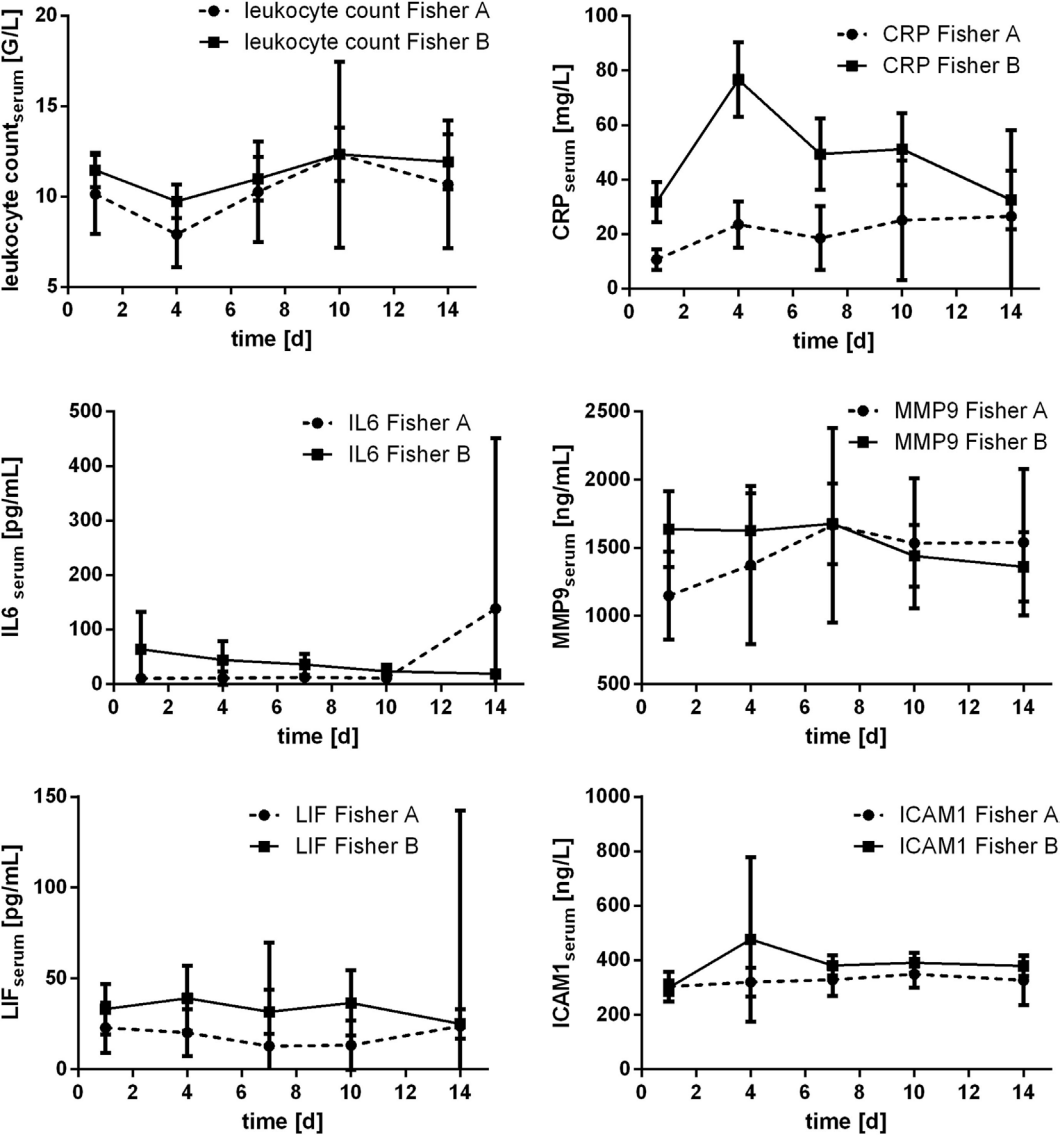
Courses of serum inflammatory parameters related to dichotomized Fisher scale score (±95% confidence interval).

Courses of inflammatory parameters additionally were compared related to Fisher scale score. Grading according to Fisher scale score was available for *n* = 81 patients [Fisher 1: *n* = 1 (1.2%); Fisher 2: *n* = 13 (16%); Fisher 3: *n* = 27 (33.3%); and Fisher 4: *n* = 40 (49.4%)]. For statistical purposes, Fisher grading was dichotomized according to severity of aSAH (Fisher A: Fisher 1 and 2; Fisher B: Fisher 3 and 4). Especially course of CRP was related to Fisher grading: the measured values significantly differed related to dichotomized Fisher scale score at day 1 (*p* = 0.019), day 4 (*p* = 0.001), and day 7 (*p* = 0.033; Mann–Whitney *U* test). Significant difference was also seen for serum IL6 at day 1 (*p* = 0.006) and day 4 (*p* = 0.002), further for serum LIF at day 7 (*p* = 0.038; Mann–Whitney *U* test). All courses are displayed in Figure 8.

### Courses of Inflammatory Parameters Related to WFNS Scale Score

Courses of inflammatory parameters also have been related to the impact of hemorrhage expressed according to WFNS scale score, which may reflect the EBI. The score was available for *n* = 81 patients [WFNS 1: *n* = 24 (29.6%); WFNS 2: *n* = 15 (18.5%); WFNS 3: *n* = 5 (6.2%); WFNS 4: *n* = 14 (7.3%); WFNS 5: *n* = 23 (28.5%)]. Again, grading scale scores were dichotomized for statistical purposes (WFNS A: WFNS 1–3; WFNS B: WFNS 4 and 5). Particularly courses of IL6 levels were related to the impact of hemorrhage expressed according to WFNS scale score: significant differences of the values for the WFNS A vs. WFNS B group were found at day 1 (*p* = 0.001); day 7 (*p* = 0.007); day 10 (*p* = 0.010), and day 14 (*p* = 0.035; Mann–Whitney *U* test). From day 1 to 10, mean IL6 values in the WFNS A group were lower than in the WFNS B group, but at day 14 it changed to higher mean values in the WFNS A group compared with WFNS B.

Levels of CRP measured early after the impact of aSAH also were found to be related to WFNS scale score: at days 1 and 4 significantly higher values were seen in the WFNS B group compared with the WFNS A group (day 1: *p* = 0.039; day 4: *p* = 0.034; Mann–Whitney *U* test).

No significant differences related to dichotomized WFNS scale score were found for leukocyte count, MMP9, LIF, and ICAM.

**Figure 9:**
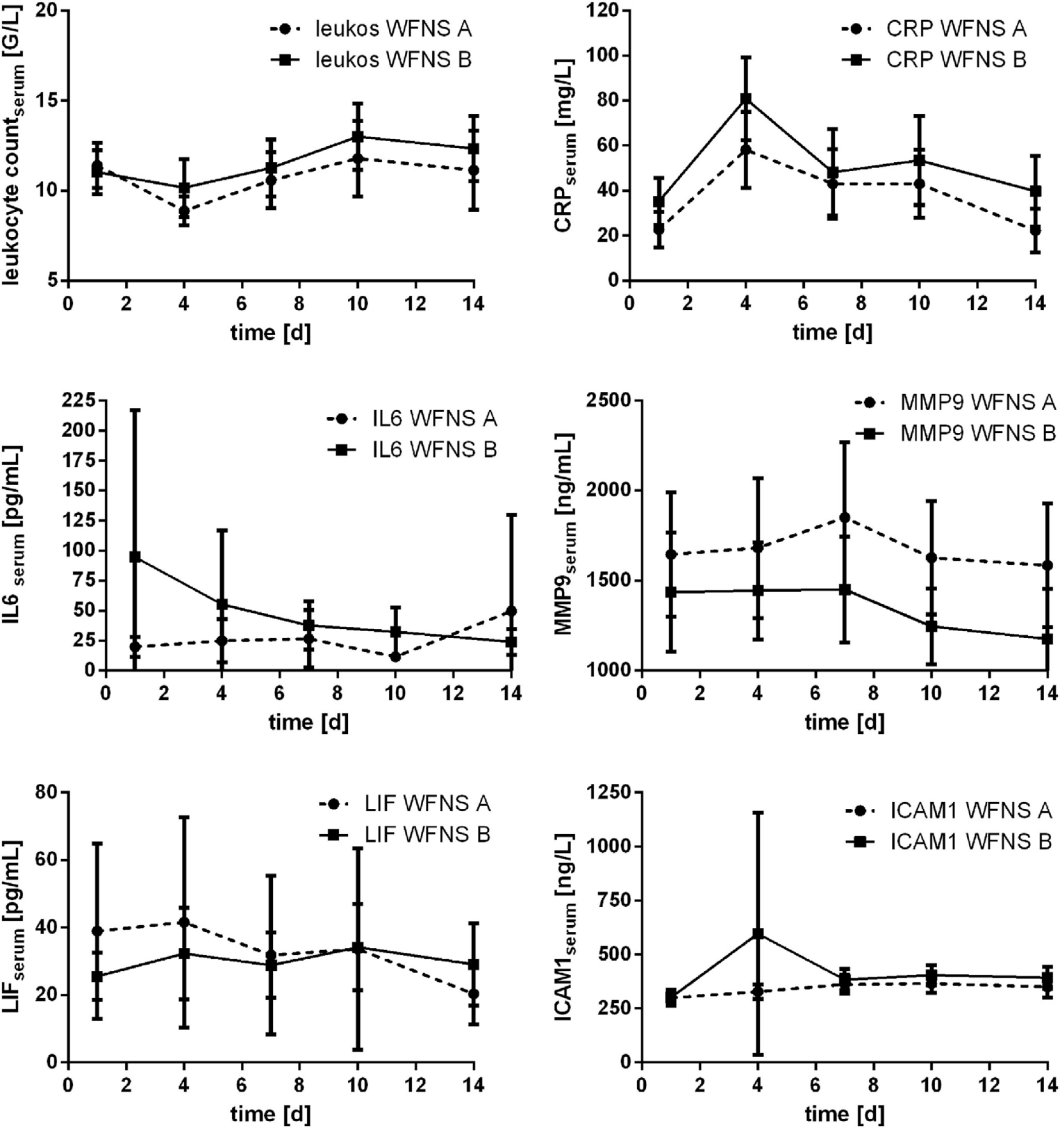
Courses of serum inflammatory parameters related to dichotomized WFNS scale score (±95% confidence interval).

All courses are displayed in Figure 9.

## Discussion

Here, we illustrate the courses of several inflammatory parameters (IL6, MMP9, ICAM1, and LIF) in CSF and serum samples after aSAH, additionally courses of CRP and leukocyte count in serum. The levels of IL6 and LIF are far higher in CSF samples than in serum samples during the entire observation period, whereas MMP9 and ICAM1 were detected in higher concentrations in serum samples. The courses of CRP and leukocyte count nearly act inversely.

Our evaluation of the measured values related to the occurrence of DCI illustrated the usual problem that the wide range of the measured values hampers their interpretation and usage as a biomarker. However, understanding the inflammatory response after aSAH and generating a database may facilitate further studies: realistic sample size calculations on the basis of a multicenter database will increase the quality and clinical relevance of the acquired results.

Interleukin 6 is a pleiotropic cytokine. Its secretion is induced by various stimuli (such as infectious disease, trauma, and other causes of tissue damage, but also during chronic conditions, e.g., depression or chronic pain). There are plenty of publications dealing with the influence of IL6 secretion after aSAH on outcome and occurrence of DCI (1, 2, 8, 10, 14, 16–19). Time courses of cytokine levels in CSF provided by us are similar to those illustrated by Niwa and colleagues (10), However, others demonstrate a later peak (1, 20). In total, schedules for sampling acquisition differ a lot among the previously mentioned studies; thus, comparison of results or courses is hampered. Anyway, wide ranges of IL6 levels are seen in all of the studies. Of note, related to WFNS scale score IL6 levels were found to be indicators for injury’s severity in our study. Nevertheless, the usage of IL6 as a biomarker is hampered by its non-specificity and its ambiguous effect showing both pro-inflammatory and destructive as well as regenerative aspects.

Higher levels of MMP9 have been associated with unfavorable outcome (21). Similar to our data, SDs (i.e., variance of measured values) are pronounced, and serum levels are more than 10-fold higher than the CSF levels.

The interpretation of ICAM1 in CSF samples is hampered by the LOD of the used assay, as the LOD was not reached in 45.8% of the samples. The ICAM1 CSF levels in our cohort are somewhat lower than those presented by Kaynar and colleagues (22), serum levels are similar. Nevertheless, comparison of time course is not possible due to different schedule for sample acquisition. Mack and colleagues also have presented ICAM1 serum levels over time after aSAH (23). Time courses seem to be similar; however, serum levels measured are slightly lower.

There are no previous data on LIF after aSAH. We demonstrate an early peak in CSF samples with far higher overall levels than in serum samples.

Leukocyte count and CRP levels have been associated with prognosis as well as vulnerability for DCI (5, 21, 24–27). Both parameters are unspecific and therefore not useful as biomarkers. Anyway, pronounced leukocytosis seems to reflect severity of disease and consecutively prognosis.

We present time courses of CSF and serum inflammatory parameters after aSAH. The results of the study are limited by the fact that time courses are displayed only descriptively. Furthermore, sample size of the presented data is quite low.

However, we estimate that mere description is useful to understand the complex inflammatory response after aSAH.

Due to their marginal specificity and wide interindividual range, inflammatory parameters hardly serve as biomarkers. Nevertheless, there is a distinct inflammatory response after aSAH, which includes a vast spectrum of parameters interacting with each other. Beneficial as well as damaging stimuli may result from the inflammatory reaction. The interactions of this reaction are poorly understood. As far as our experience goes, we hold that further illustration of courses and possible interactions of inflammatory reaction after aSAH is essential before associations with outcome or occurrence of DCI in search for a biomarker are promising. Generally, setting up multicenter databases to further illustrate and hopefully understand inflammatory response after aSAH has more prospect of success than searching for a single biomarker resulting in controversial, clinically irrelevant results. Furthermore, these data may lay the groundwork for further studies’ realistic sample size calculations to create clinically relevant and reliable results. Thus, we encourage everyone involved in research of inflammation after aSAH to cooperate and build up networks to share acquired data.

## Ethics Statement

This study was carried out in accordance with the recommendations of “Ethikkommission an der Medizinischen Fakultät der Rheinischen Friedrich-Wilhelms Universität Bonn name of guidelines, name of committee” with written informed consent from all subjects. All subjects gave written informed consent in accordance with the Declaration of Helsinki. The protocol was approved by the local ethics committee (Ethikkommission an der Medizinischen Fakultät der Rheinischen Friedrich-Wilhelms-Universität Bonn, Germany; EK 199/08).

## Author Contributions

AH conceived the study, analyzed the data, and wrote the preliminary draft. BS-W organized and supervised analysis of serum and CSF samples; assisted in interpretation of data. MV and HC participated in the analyses and helped to draft the manuscript. GS helped with data analyses and assisted the revision of the manuscript. MC participated in the design of the study and coordination and helped to draft the manuscript. All the authors read, revised, and approved the final manuscript.

## Conflict of Interest Statement

The authors declare that the research was conducted in the absence of any commercial or financial relationships that could be construed as a potential conflict of interest.

## Supplementary Material

Table S1 Baseline characteristics of patients included.
