# Supplementary material for: Time Courses of Inflammatory Markers after Aneurysmal Subarachnoid Hemorrhage and Their Possible Relevance for Future Studies
Source: Front Neurol. 2017 Dec 22;8:694. doi: 10.3389/fneur.2017.00694 (PMC5744005; doi:10.3389/fneur.2017.00694)
Supplement: Table S1 — Baseline characteristics of patients included. [file Table_1.docx]

| **Age** | 53.8 ± 13.2 |
| --- | --- |
| **Sex** | Female: n = 51 (63%) |
|  | Male: n = 30 (37%) |
| **Treatment** | Coiling: n = 55 (68%) |
|  | Clipping: n =26 (32%) |
| **WFNS scale score** | WFNS 1: n = 24 (30%) |
|  | WFNS 2: n = 15 (19%) |
|  | WFNS 3: n = 5 (6%) |
|  | WFNS 4: n = 14 (17%) |
|  | WFNS 5: n = 23 (28%) |
| **Aneurysm location** | MCA: n = 17 (21%) |
|  | ACOM: n = 26 (32%) |
|  | PCOM: n = 19 (24%) |
|  | BA: n = 7 (9%) |
|  | VA: n = 3 (4%) |
|  | Other: n = 3 (4%) |
|  | Not given: n = 6 (7%) |
| **Fisher score** | Fisher 1: n = 1 (1%) |
|  | Fisher 2: n = 13 (16%) |
|  | Fisher 3: n = 27 (33%) |
|  | Fisher 4: n = 40 (49%) |
| **DCI** | Positive: n = 18 (22%) |
| **Infection** | Day 1: n = 8 (10%) |
|  | Day 4: n = 27 (33%) |
|  | Day 7: n = 38 (47%) |
|  | Day 10: n = 36 ( 44%) |
|  | Day 14: n 0 26 (32%) |
